# Crystal structure of [2,6-bis(adamantan-1-yl)-4-*tert*-butylphenolato-κ*O*]dimethylaluminium(III)

**DOI:** 10.1107/S1600536814020492

**Published:** 2014-09-24

**Authors:** Lei Wang, Li Yang

**Affiliations:** a College of Chemistry and Chemical Engineering, Northwest Normal University, Lanzhou, 730070, People’s Republic of China

**Keywords:** crystal structure, aluminium, C_2_O trigonal coordination geometry, adamantyl-substituted phenol ligand

## Abstract

The title compound, [Al(CH_3_)_2_(C_30_H_41_O)] is synthesized by the reaction of 2,6-di-adamantyl-4-*tert*-butyl-phenol with Al(CH_3_)_3_ in a nitro­gen atmosphere. In the mol­ecule, the coordination geometry around the Al^III^ atom is slightly distorted C_2_O trigonal (the sum of the bond angles subtended at Al atom being 359.9°), which is rarely reported for organometallic aluminium compounds. The coordination plane is approximately perpendicular to the benzene ring [the dihedral angle = 87.73 (16)°]. There is no inter­molecular hydrogen bonding in the crystal structure.

## Related literature   

For metal complexes with an adamantyl-substituted phenol ligand, see: Watanabe *et al.* (2010 ▶); Hatanaka *et al.* (2011 ▶). For applications of aluminium alkyl compounds in catalysis for ring-opening polymerization of cyclic esters, see: Liu *et al.* (2001 ▶). For related structures with three-coordinate aluminium, see: Jerius *et al.* (1986 ▶); Klis *et al.* (2011 ▶).
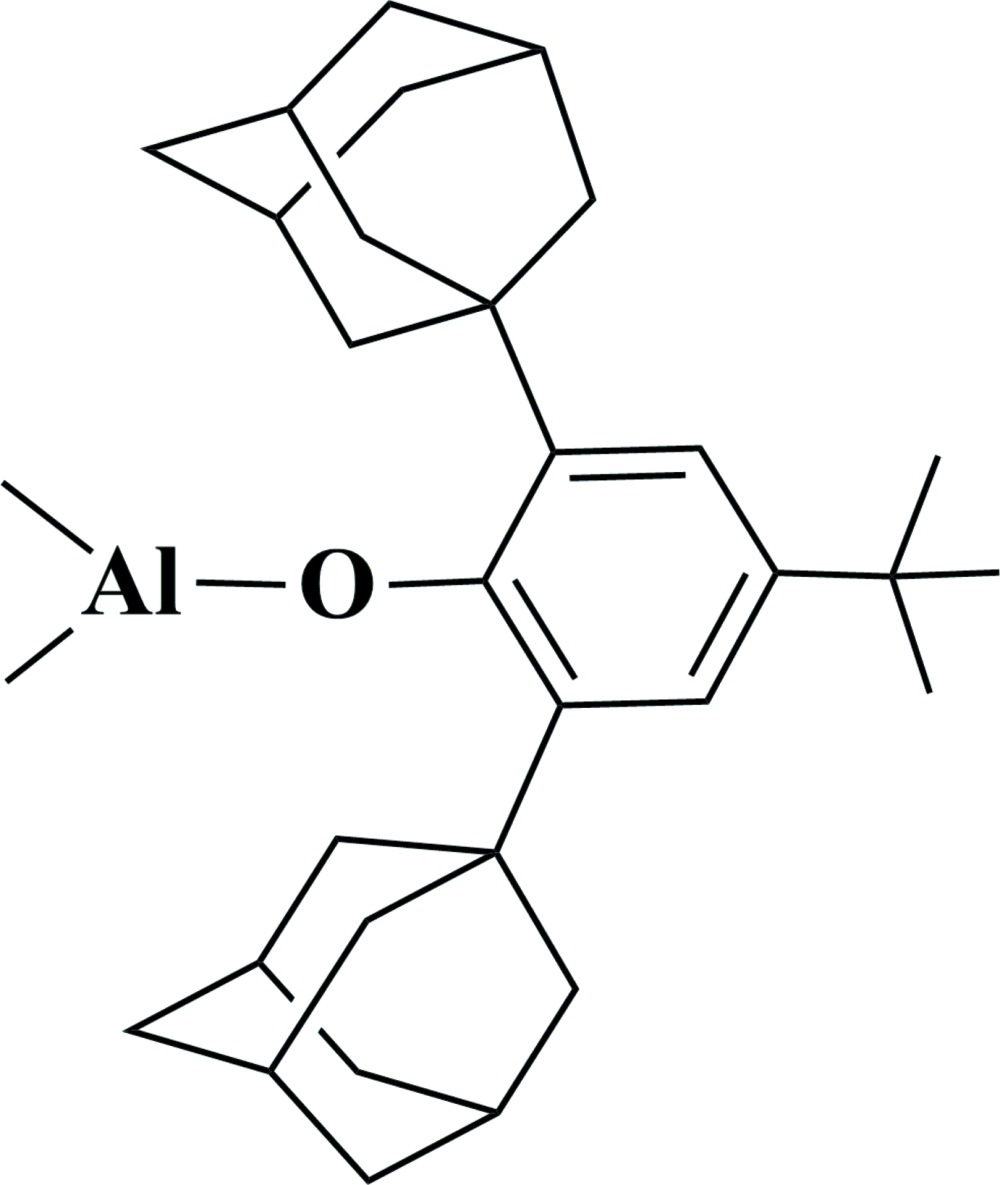


## Experimental   

### Crystal data  

[Al(CH_3_)_2_(C_30_H_41_O)]
*M* *_r_* = 474.68
Monoclinic, 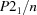

*a* = 7.266 (4) Å
*b* = 20.93 (1) Å
*c* = 18.200 (9) Å
β = 95.038 (6)°
*V* = 2757 (2) Å^3^
*Z* = 4
Mo *K*α radiation
μ = 0.10 mm^−1^
*T* = 293 K
0.35 × 0.29 × 0.21 mm

### Data collection  

Bruker SMART 1000 diffractometer
14027 measured reflections
4930 independent reflections
2380 reflections with *I* > 2σ(*I*)
*R*_int_ = 0.091

### Refinement  

*R*[*F* ^2^ > 2σ(*F* ^2^)] = 0.080
*wR*(*F* ^2^) = 0.219
*S* = 1.00
4930 reflections
312 parameters
H-atom parameters constrained
Δρ_max_ = 0.29 e Å^−3^
Δρ_min_ = −0.28 e Å^−3^

### 

Data collection: *SMART* (Bruker, 2004 ▶); cell refinement: *SAINT* (Bruker, 2004 ▶); data reduction: *SAINT*; program(s) used to solve structure: *SHELXS97* (Sheldrick, 2008 ▶); program(s) used to refine structure: *SHELXL97* (Sheldrick, 2008 ▶); molecular graphics: *SHELXTL* (Sheldrick, 2008 ▶); software used to prepare material for publication: *publCIF* (Westrip, 2010 ▶).

## Supplementary Material

Crystal structure: contains datablock(s) I, global. DOI: 10.1107/S1600536814020492/xu5813sup1.cif

Structure factors: contains datablock(s) I. DOI: 10.1107/S1600536814020492/xu5813Isup2.hkl

**Fig. 1.**
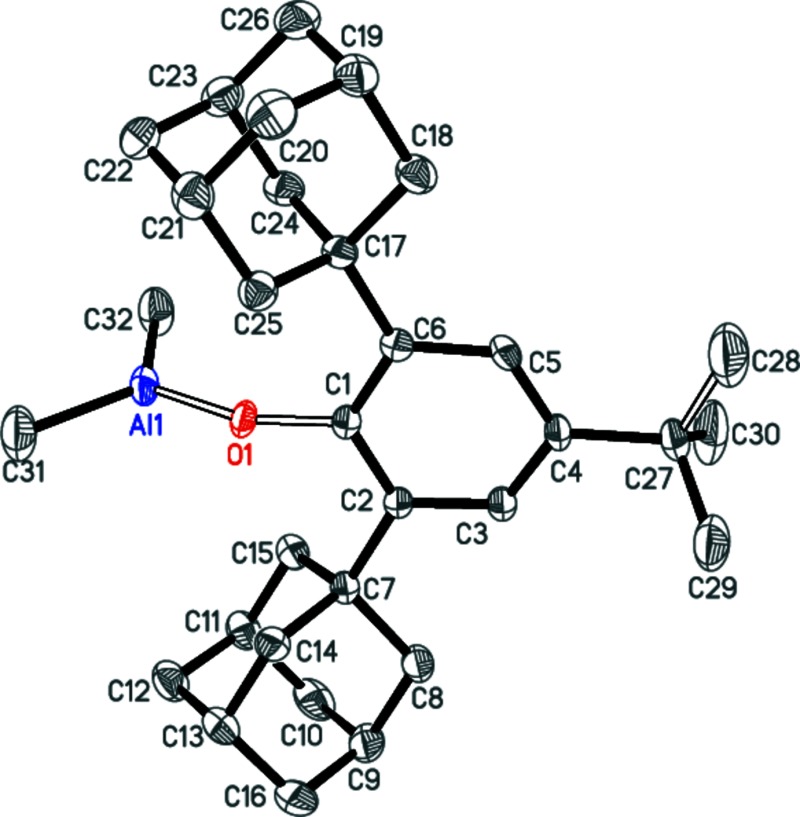
An ORTEP view of the molecular structure of the title molecule with the atom-numbering. Displacement ellipsoids are drawn at 30%. The H atoms have been omitted for clarity.

**Fig. 2.**
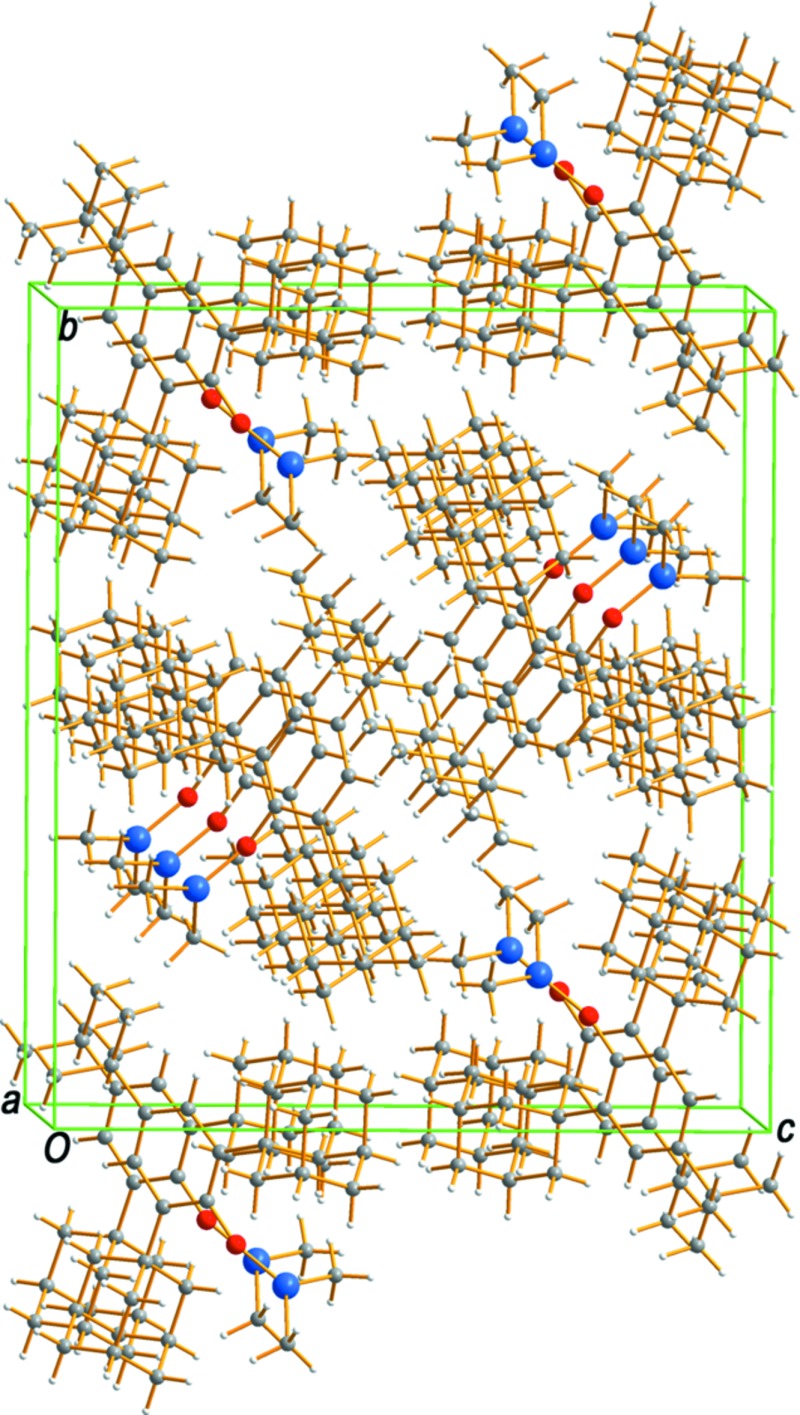
Molecular packing of the title compound viewed along a axis.

ORTEP . DOI: 10.1107/S1600536814020492/xu5813fig1.tif
An *ORTEP* view of the mol­ecular structure of the title mol­ecule with the atom-numbering. Displacement ellipsoids are drawn at 30%. The H atoms have been omitted for clarity.

a . DOI: 10.1107/S1600536814020492/xu5813fig2.tif
Mol­ecular packing of the title compound viewed along *a* axis.

CCDC reference: 1019044

Additional supporting information:  crystallographic information; 3D view; checkCIF report

## supplementary crystallographic information

### S1. Experimental

A hexane solution of Al(CH_3_)_3_ (1.0 M, 1.1 mL, 1.1 mmol) was added dropwise to 2,6-di-adamantyl-4-*tert*-butyl-phenol (0.46g, 1.0 mmol) in THF (20 mL) at 273 K with stirring. The mixture was warmed up to 313 K and stirred for 4 h, and then the solution was filtered through celite. The filtrate was concentrated to ca. 10 mL and left at room temperature overnight, colorless crystals were obtained. The single crystals were gathered and isolated for studies. Yield: 0.29g (61%). ^1^H NMR (298 K, CDCl_3_, 300 MHz, ppm): δ 7.11 (s, 2H, Ar*H*), 2.16 (s, 12H, adamantyl), 2.03 (s, 6H, adamantyl), 1.78 (s, 12H, adamantyl), 1.28 (s, 9H, C(C*H*_3_)_3_), -0.65 (s, 6H, (C*H*_3_)_2_).

### S2. Refinement

H atoms were fixed geometrically and treated as riding with C–H = 0.93–0.97 Å, U_iso_(H) = 1.5U_eq_(C) for methyl H atoms and 1.2U_eq_(C) for the others.

### Crystal data

**Table:**

[Al(CH_3_)_2_(C_30_H_41_O)]	*F*(000) = 1040
*M**_r_* = 474.68	*D*_x_ = 1.144 Mg m^−^^3^
Monoclinic, *P*2_1_/*n*	Mo *K*α radiation, λ = 0.71073 Å
Hall symbol: -P 2yn	Cell parameters from 4930 reflections
*a* = 7.266 (4) Å	θ = 2.2–25.2°
*b* = 20.93 (1) Å	µ = 0.10 mm^−^^1^
*c* = 18.200 (9) Å	*T* = 293 K
β = 95.038 (6)°	Block, colorless
*V* = 2757 (2) Å^3^	0.35 × 0.29 × 0.21 mm
*Z* = 4	

### Data collection

**Table:**

Bruker SMART 1000 diffractometer	2380 reflections with *I* > 2σ(*I*)
Radiation source: fine-focus sealed tube	*R*_int_ = 0.091
Graphite monochromator	θ_max_ = 25.2°, θ_min_ = 2.3°
phi and ω scans	*h* = −8→8
14027 measured reflections	*k* = −22→25
4930 independent reflections	*l* = −21→18

### Refinement

**Table:**

Refinement on *F*^2^	Primary atom site location: structure-invariant direct methods
Least-squares matrix: full	Secondary atom site location: difference Fourier map
*R*[*F*^2^ > 2σ(*F*^2^)] = 0.080	Hydrogen site location: inferred from neighbouring sites
*wR*(*F*^2^) = 0.219	H-atom parameters constrained
*S* = 1.00	*w* = 1/[σ^2^(*F*_o_^2^) + (0.104*P*)^2^] where *P* = (*F*_o_^2^ + 2*F*_c_^2^)/3
4930 reflections	(Δ/σ)_max_ = 0.001
312 parameters	Δρ_max_ = 0.29 e Å^−^^3^
0 restraints	Δρ_min_ = −0.28 e Å^−^^3^

### Special details

**Table:**

Geometry. All esds (except the esd in the dihedral angle between two l.s. planes) are estimated using the full covariance matrix. The cell esds are taken into account individually in the estimation of esds in distances, angles and torsion angles; correlations between esds in cell parameters are only used when they are defined by crystal symmetry. An approximate (isotropic) treatment of cell esds is used for estimating esds involving l.s. planes.
Refinement. Refinement of F^2^ against ALL reflections. The weighted R-factor wR and goodness of fit S are based on F^2^, conventional R-factors R are based on F, with F set to zero for negative F^2^. The threshold expression of F^2^ > 2sigma(F^2^) is used only for calculating R-factors(gt) etc. and is not relevant to the choice of reflections for refinement. R-factors based on F^2^ are statistically about twice as large as those based on F, and R- factors based on ALL data will be even larger.

### Fractional atomic coordinates and isotropic or equivalent isotropic displacement parameters (Å^2^)

**Table:**

	*x*	*y*	*z*	*U*_iso_*/*U*_eq_	
Al1	0.97063 (19)	0.19082 (6)	0.67409 (7)	0.0430 (4)	
O1	0.9966 (4)	0.13995 (12)	0.74514 (14)	0.0392 (8)	
C1	0.9359 (5)	0.08875 (19)	0.7833 (2)	0.0320 (10)	
C2	0.8177 (5)	0.09989 (18)	0.8396 (2)	0.0298 (9)	
C3	0.7622 (6)	0.04657 (19)	0.8780 (2)	0.0370 (11)	
H3	0.6822	0.0529	0.9145	0.044*	
C4	0.8187 (6)	−0.01472 (19)	0.8651 (2)	0.0357 (10)	
C5	0.9352 (6)	−0.02303 (18)	0.8104 (2)	0.0349 (10)	
H5	0.9733	−0.0643	0.8004	0.042*	
C6	1.0004 (5)	0.02757 (18)	0.7687 (2)	0.0310 (10)	
C7	0.7586 (5)	0.16811 (19)	0.8619 (2)	0.0341 (10)	
C8	0.6387 (7)	0.1659 (2)	0.9279 (2)	0.0482 (12)	
H8A	0.5286	0.1408	0.9147	0.058*	
H8B	0.7074	0.1452	0.9694	0.058*	
C9	0.5826 (7)	0.2328 (2)	0.9507 (3)	0.0600 (15)	
H9	0.5062	0.2296	0.9923	0.072*	
C10	0.4721 (7)	0.2658 (2)	0.8862 (3)	0.0622 (15)	
H10A	0.3603	0.2418	0.8722	0.075*	
H10B	0.4372	0.3084	0.9006	0.075*	
C11	0.5904 (6)	0.2695 (2)	0.8215 (3)	0.0456 (12)	
H11	0.5204	0.2903	0.7797	0.055*	
C12	0.7641 (7)	0.3084 (2)	0.8445 (3)	0.0569 (13)	
H12A	0.8395	0.3119	0.8033	0.068*	
H12B	0.7295	0.3512	0.8586	0.068*	
C13	0.8742 (6)	0.2759 (2)	0.9094 (3)	0.0498 (13)	
H13	0.9859	0.3008	0.9235	0.060*	
C14	0.9288 (6)	0.20866 (19)	0.8875 (2)	0.0395 (11)	
H14A	0.9963	0.1881	0.9293	0.047*	
H14B	1.0098	0.2113	0.8480	0.047*	
C15	0.6429 (6)	0.20245 (19)	0.7997 (2)	0.0397 (11)	
H15A	0.7123	0.2046	0.7567	0.048*	
H15B	0.5314	0.1780	0.7866	0.048*	
C16	0.7573 (8)	0.2720 (2)	0.9743 (3)	0.0655 (15)	
H16A	0.7231	0.3146	0.9892	0.079*	
H16B	0.8267	0.2516	1.0158	0.079*	
C17	1.1379 (5)	0.01391 (19)	0.7105 (2)	0.0329 (10)	
C18	1.1905 (6)	−0.0569 (2)	0.7070 (3)	0.0462 (12)	
H18A	1.2437	−0.0706	0.7551	0.055*	
H18B	1.0800	−0.0820	0.6945	0.055*	
C19	1.3288 (6)	−0.0693 (2)	0.6500 (3)	0.0518 (13)	
H19	1.3590	−0.1149	0.6494	0.062*	
C20	1.5037 (6)	−0.0310 (2)	0.6697 (3)	0.0574 (14)	
H20A	1.5911	−0.0384	0.6333	0.069*	
H20B	1.5604	−0.0446	0.7174	0.069*	
C21	1.4561 (6)	0.0396 (2)	0.6721 (3)	0.0514 (13)	
H21	1.5685	0.0646	0.6849	0.062*	
C22	1.3677 (7)	0.0605 (2)	0.5973 (3)	0.0540 (13)	
H22A	1.4557	0.0555	0.5606	0.065*	
H22B	1.3345	0.1053	0.5992	0.065*	
C23	1.1952 (6)	0.0208 (2)	0.5751 (2)	0.0489 (12)	
H23	1.1412	0.0340	0.5262	0.059*	
C24	1.0574 (6)	0.0319 (2)	0.6321 (2)	0.0421 (11)	
H24A	0.9474	0.0066	0.6191	0.050*	
H24B	1.0215	0.0766	0.6313	0.050*	
C25	1.3198 (6)	0.0507 (2)	0.7292 (2)	0.0407 (11)	
H25A	1.2933	0.0960	0.7320	0.049*	
H25B	1.3748	0.0371	0.7772	0.049*	
C26	1.2439 (7)	−0.0492 (2)	0.5743 (3)	0.0574 (14)	
H26A	1.3308	−0.0570	0.5378	0.069*	
H26B	1.1336	−0.0743	0.5610	0.069*	
C27	0.7524 (7)	−0.0707 (2)	0.9104 (3)	0.0462 (12)	
C28	0.8381 (12)	−0.1335 (3)	0.8917 (4)	0.144 (4)	
H28A	0.9703	−0.1300	0.8983	0.216*	
H28B	0.8008	−0.1443	0.8413	0.216*	
H28C	0.7979	−0.1663	0.9235	0.216*	
C29	0.7941 (9)	−0.0586 (3)	0.9918 (3)	0.085 (2)	
H29A	0.7373	−0.0912	1.0193	0.128*	
H29B	0.7463	−0.0176	1.0041	0.128*	
H29C	0.9254	−0.0593	1.0039	0.128*	
C30	0.5433 (8)	−0.0774 (3)	0.8967 (3)	0.095 (2)	
H30A	0.5012	−0.1101	0.9282	0.143*	
H30B	0.5114	−0.0889	0.8461	0.143*	
H30C	0.4857	−0.0376	0.9071	0.143*	
C31	1.1617 (7)	0.2553 (2)	0.6788 (3)	0.0731 (17)	
H31A	1.1611	0.2785	0.7244	0.110*	
H31B	1.1390	0.2843	0.6382	0.110*	
H31C	1.2797	0.2353	0.6763	0.110*	
C32	0.7745 (7)	0.1835 (2)	0.5967 (3)	0.0630 (15)	
H32A	0.7100	0.1439	0.6019	0.095*	
H32B	0.8253	0.1843	0.5497	0.095*	
H32C	0.6902	0.2185	0.5996	0.095*	

### Atomic displacement parameters (Å^2^)

**Table:**

	*U*^11^	*U*^22^	*U*^33^	*U*^12^	*U*^13^	*U*^23^
Al1	0.0514 (9)	0.0344 (8)	0.0441 (8)	−0.0058 (7)	0.0088 (7)	0.0118 (6)
O1	0.0512 (19)	0.0300 (16)	0.0392 (17)	−0.0003 (14)	0.0191 (14)	0.0105 (13)
C1	0.033 (2)	0.031 (2)	0.032 (2)	−0.0034 (19)	0.0030 (19)	0.0045 (18)
C2	0.032 (2)	0.029 (2)	0.029 (2)	−0.0009 (18)	0.0062 (19)	0.0022 (18)
C3	0.043 (3)	0.031 (2)	0.039 (3)	−0.002 (2)	0.010 (2)	0.0022 (19)
C4	0.040 (3)	0.027 (2)	0.040 (3)	−0.006 (2)	0.007 (2)	0.006 (2)
C5	0.041 (3)	0.020 (2)	0.043 (3)	0.0025 (19)	0.006 (2)	0.0010 (19)
C6	0.028 (2)	0.032 (2)	0.033 (2)	0.0003 (18)	0.0012 (19)	−0.0025 (19)
C7	0.037 (2)	0.031 (2)	0.035 (2)	−0.0041 (19)	0.009 (2)	0.0008 (18)
C8	0.058 (3)	0.034 (3)	0.056 (3)	−0.002 (2)	0.022 (3)	0.000 (2)
C9	0.077 (4)	0.043 (3)	0.066 (4)	0.001 (3)	0.043 (3)	−0.005 (3)
C10	0.051 (3)	0.035 (3)	0.103 (4)	0.009 (2)	0.019 (3)	−0.012 (3)
C11	0.039 (3)	0.034 (3)	0.064 (3)	0.004 (2)	0.004 (2)	0.002 (2)
C12	0.067 (3)	0.032 (3)	0.072 (3)	0.000 (3)	0.009 (3)	0.002 (2)
C13	0.053 (3)	0.041 (3)	0.054 (3)	−0.014 (2)	−0.002 (3)	−0.007 (2)
C14	0.040 (3)	0.042 (3)	0.036 (3)	−0.002 (2)	0.002 (2)	−0.004 (2)
C15	0.034 (2)	0.037 (3)	0.049 (3)	0.000 (2)	0.004 (2)	−0.002 (2)
C16	0.102 (4)	0.040 (3)	0.055 (3)	0.000 (3)	0.013 (3)	−0.013 (2)
C17	0.034 (2)	0.032 (2)	0.033 (2)	0.0006 (19)	0.0028 (19)	−0.0046 (19)
C18	0.049 (3)	0.035 (3)	0.056 (3)	0.003 (2)	0.011 (2)	−0.002 (2)
C19	0.048 (3)	0.040 (3)	0.070 (4)	0.011 (2)	0.018 (3)	−0.004 (3)
C20	0.040 (3)	0.062 (3)	0.072 (4)	0.007 (3)	0.012 (3)	0.000 (3)
C21	0.036 (3)	0.055 (3)	0.063 (3)	−0.009 (2)	0.007 (2)	−0.002 (3)
C22	0.053 (3)	0.056 (3)	0.057 (3)	−0.006 (3)	0.024 (3)	−0.005 (3)
C23	0.049 (3)	0.060 (3)	0.038 (3)	−0.004 (3)	0.006 (2)	−0.001 (2)
C24	0.038 (3)	0.044 (3)	0.044 (3)	0.005 (2)	0.004 (2)	−0.007 (2)
C25	0.041 (3)	0.041 (3)	0.040 (3)	0.004 (2)	0.007 (2)	0.001 (2)
C26	0.055 (3)	0.060 (4)	0.060 (3)	−0.006 (3)	0.022 (3)	−0.015 (3)
C27	0.061 (3)	0.029 (3)	0.049 (3)	−0.008 (2)	0.007 (2)	0.009 (2)
C28	0.252 (10)	0.036 (4)	0.166 (7)	0.029 (5)	0.143 (8)	0.035 (4)
C29	0.124 (5)	0.062 (4)	0.068 (4)	−0.030 (4)	−0.002 (4)	0.028 (3)
C30	0.086 (5)	0.102 (5)	0.095 (5)	−0.048 (4)	−0.007 (4)	0.049 (4)
C31	0.084 (4)	0.065 (4)	0.070 (4)	−0.027 (3)	0.005 (3)	0.020 (3)
C32	0.067 (4)	0.058 (3)	0.063 (3)	−0.009 (3)	−0.002 (3)	0.016 (3)

### Geometric parameters (Å, º)

**Table:**

Al1—O1	1.673 (3)	C17—C25	1.541 (5)
Al1—C32	1.920 (5)	C18—C19	1.528 (6)
Al1—C31	1.933 (5)	C18—H18A	0.9700
O1—C1	1.371 (4)	C18—H18B	0.9700
C1—C6	1.397 (5)	C19—C26	1.518 (6)
C1—C2	1.412 (5)	C19—C20	1.518 (6)
C2—C3	1.395 (5)	C19—H19	0.9800
C2—C7	1.555 (5)	C20—C21	1.520 (6)
C3—C4	1.373 (5)	C20—H20A	0.9700
C3—H3	0.9300	C20—H20B	0.9700
C4—C5	1.372 (5)	C21—C25	1.515 (6)
C4—C27	1.535 (5)	C21—C22	1.518 (6)
C5—C6	1.409 (5)	C21—H21	0.9800
C5—H5	0.9300	C22—C23	1.528 (6)
C6—C17	1.546 (5)	C22—H22A	0.9700
C7—C15	1.528 (5)	C22—H22B	0.9700
C7—C14	1.538 (5)	C23—C26	1.509 (6)
C7—C8	1.547 (5)	C23—C24	1.521 (6)
C8—C9	1.526 (6)	C23—H23	0.9800
C8—H8A	0.9700	C24—H24A	0.9700
C8—H8B	0.9700	C24—H24B	0.9700
C9—C10	1.527 (7)	C25—H25A	0.9700
C9—C16	1.540 (7)	C25—H25B	0.9700
C9—H9	0.9800	C26—H26A	0.9700
C10—C11	1.519 (6)	C26—H26B	0.9700
C10—H10A	0.9700	C27—C28	1.507 (7)
C10—H10B	0.9700	C27—C29	1.508 (7)
C11—C15	1.516 (6)	C27—C30	1.524 (7)
C11—C12	1.530 (6)	C28—H28A	0.9600
C11—H11	0.9800	C28—H28B	0.9600
C12—C13	1.527 (6)	C28—H28C	0.9600
C12—H12A	0.9700	C29—H29A	0.9600
C12—H12B	0.9700	C29—H29B	0.9600
C13—C16	1.517 (6)	C29—H29C	0.9600
C13—C14	1.525 (6)	C30—H30A	0.9600
C13—H13	0.9800	C30—H30B	0.9600
C14—H14A	0.9700	C30—H30C	0.9600
C14—H14B	0.9700	C31—H31A	0.9600
C15—H15A	0.9700	C31—H31B	0.9600
C15—H15B	0.9700	C31—H31C	0.9600
C16—H16A	0.9700	C32—H32A	0.9600
C16—H16B	0.9700	C32—H32B	0.9600
C17—C18	1.533 (5)	C32—H32C	0.9600
C17—C24	1.541 (6)		
			
O1—Al1—C32	122.83 (19)	C19—C18—H18A	109.2
O1—Al1—C31	112.2 (2)	C17—C18—H18A	109.2
C32—Al1—C31	124.9 (2)	C19—C18—H18B	109.2
C1—O1—Al1	150.4 (3)	C17—C18—H18B	109.2
O1—C1—C6	119.5 (4)	H18A—C18—H18B	107.9
O1—C1—C2	118.8 (3)	C26—C19—C20	109.5 (4)
C6—C1—C2	121.6 (4)	C26—C19—C18	109.2 (4)
C3—C2—C1	116.9 (4)	C20—C19—C18	109.6 (4)
C3—C2—C7	120.3 (3)	C26—C19—H19	109.5
C1—C2—C7	122.7 (3)	C20—C19—H19	109.5
C4—C3—C2	123.9 (4)	C18—C19—H19	109.5
C4—C3—H3	118.0	C19—C20—C21	109.4 (4)
C2—C3—H3	118.0	C19—C20—H20A	109.8
C5—C4—C3	117.0 (4)	C21—C20—H20A	109.8
C5—C4—C27	122.4 (4)	C19—C20—H20B	109.8
C3—C4—C27	120.6 (4)	C21—C20—H20B	109.8
C4—C5—C6	123.6 (4)	H20A—C20—H20B	108.2
C4—C5—H5	118.2	C25—C21—C22	108.7 (4)
C6—C5—H5	118.2	C25—C21—C20	109.4 (4)
C1—C6—C5	116.9 (4)	C22—C21—C20	109.5 (4)
C1—C6—C17	123.2 (3)	C25—C21—H21	109.7
C5—C6—C17	119.9 (3)	C22—C21—H21	109.7
C15—C7—C14	109.9 (3)	C20—C21—H21	109.7
C15—C7—C8	106.1 (3)	C21—C22—C23	110.7 (4)
C14—C7—C8	105.9 (3)	C21—C22—H22A	109.5
C15—C7—C2	112.7 (3)	C23—C22—H22A	109.5
C14—C7—C2	110.7 (3)	C21—C22—H22B	109.5
C8—C7—C2	111.3 (3)	C23—C22—H22B	109.5
C9—C8—C7	111.5 (4)	H22A—C22—H22B	108.1
C9—C8—H8A	109.3	C26—C23—C24	108.9 (4)
C7—C8—H8A	109.3	C26—C23—C22	110.1 (4)
C9—C8—H8B	109.3	C24—C23—C22	108.1 (4)
C7—C8—H8B	109.3	C26—C23—H23	109.9
H8A—C8—H8B	108.0	C24—C23—H23	109.9
C8—C9—C10	109.9 (4)	C22—C23—H23	109.9
C8—C9—C16	109.3 (4)	C23—C24—C17	112.0 (4)
C10—C9—C16	109.8 (4)	C23—C24—H24A	109.2
C8—C9—H9	109.3	C17—C24—H24A	109.2
C10—C9—H9	109.3	C23—C24—H24B	109.2
C16—C9—H9	109.3	C17—C24—H24B	109.2
C11—C10—C9	108.9 (4)	H24A—C24—H24B	107.9
C11—C10—H10A	109.9	C21—C25—C17	111.8 (3)
C9—C10—H10A	109.9	C21—C25—H25A	109.3
C11—C10—H10B	109.9	C17—C25—H25A	109.3
C9—C10—H10B	109.9	C21—C25—H25B	109.3
H10A—C10—H10B	108.3	C17—C25—H25B	109.3
C15—C11—C10	109.1 (4)	H25A—C25—H25B	107.9
C15—C11—C12	110.2 (4)	C23—C26—C19	109.7 (4)
C10—C11—C12	108.9 (4)	C23—C26—H26A	109.7
C15—C11—H11	109.5	C19—C26—H26A	109.7
C10—C11—H11	109.5	C23—C26—H26B	109.7
C12—C11—H11	109.5	C19—C26—H26B	109.7
C13—C12—C11	109.7 (4)	H26A—C26—H26B	108.2
C13—C12—H12A	109.7	C28—C27—C29	108.5 (5)
C11—C12—H12A	109.7	C28—C27—C30	108.0 (5)
C13—C12—H12B	109.7	C29—C27—C30	106.7 (5)
C11—C12—H12B	109.7	C28—C27—C4	112.9 (4)
H12A—C12—H12B	108.2	C29—C27—C4	110.8 (4)
C16—C13—C14	109.3 (4)	C30—C27—C4	109.7 (4)
C16—C13—C12	109.6 (4)	C27—C28—H28A	109.5
C14—C13—C12	109.9 (4)	C27—C28—H28B	109.5
C16—C13—H13	109.4	H28A—C28—H28B	109.5
C14—C13—H13	109.4	C27—C28—H28C	109.5
C12—C13—H13	109.4	H28A—C28—H28C	109.5
C13—C14—C7	111.6 (4)	H28B—C28—H28C	109.5
C13—C14—H14A	109.3	C27—C29—H29A	109.5
C7—C14—H14A	109.3	C27—C29—H29B	109.5
C13—C14—H14B	109.3	H29A—C29—H29B	109.5
C7—C14—H14B	109.3	C27—C29—H29C	109.5
H14A—C14—H14B	108.0	H29A—C29—H29C	109.5
C11—C15—C7	112.1 (3)	H29B—C29—H29C	109.5
C11—C15—H15A	109.2	C27—C30—H30A	109.5
C7—C15—H15A	109.2	C27—C30—H30B	109.5
C11—C15—H15B	109.2	H30A—C30—H30B	109.5
C7—C15—H15B	109.2	C27—C30—H30C	109.5
H15A—C15—H15B	107.9	H30A—C30—H30C	109.5
C13—C16—C9	108.4 (4)	H30B—C30—H30C	109.5
C13—C16—H16A	110.0	Al1—C31—H31A	109.5
C9—C16—H16A	110.0	Al1—C31—H31B	109.5
C13—C16—H16B	110.0	H31A—C31—H31B	109.5
C9—C16—H16B	110.0	Al1—C31—H31C	109.5
H16A—C16—H16B	108.4	H31A—C31—H31C	109.5
C18—C17—C24	105.9 (3)	H31B—C31—H31C	109.5
C18—C17—C25	106.3 (3)	Al1—C32—H32A	109.5
C24—C17—C25	109.3 (3)	Al1—C32—H32B	109.5
C18—C17—C6	112.7 (3)	H32A—C32—H32B	109.5
C24—C17—C6	112.0 (3)	Al1—C32—H32C	109.5
C25—C17—C6	110.4 (3)	H32A—C32—H32C	109.5
C19—C18—C17	112.0 (4)	H32B—C32—H32C	109.5
