# Supplementary figures and images for: Crystal structure of [2,6-bis(adamantan-1-yl)-4-tert-butylphenolato-κO]dimethylaluminium(III)
Source: Acta Crystallogr Sect E Struct Rep Online. 2014 Sep 24;70(Pt 10):m352. doi: 10.1107/S1600536814020492 (PMC4257224; doi:10.1107/S1600536814020492)

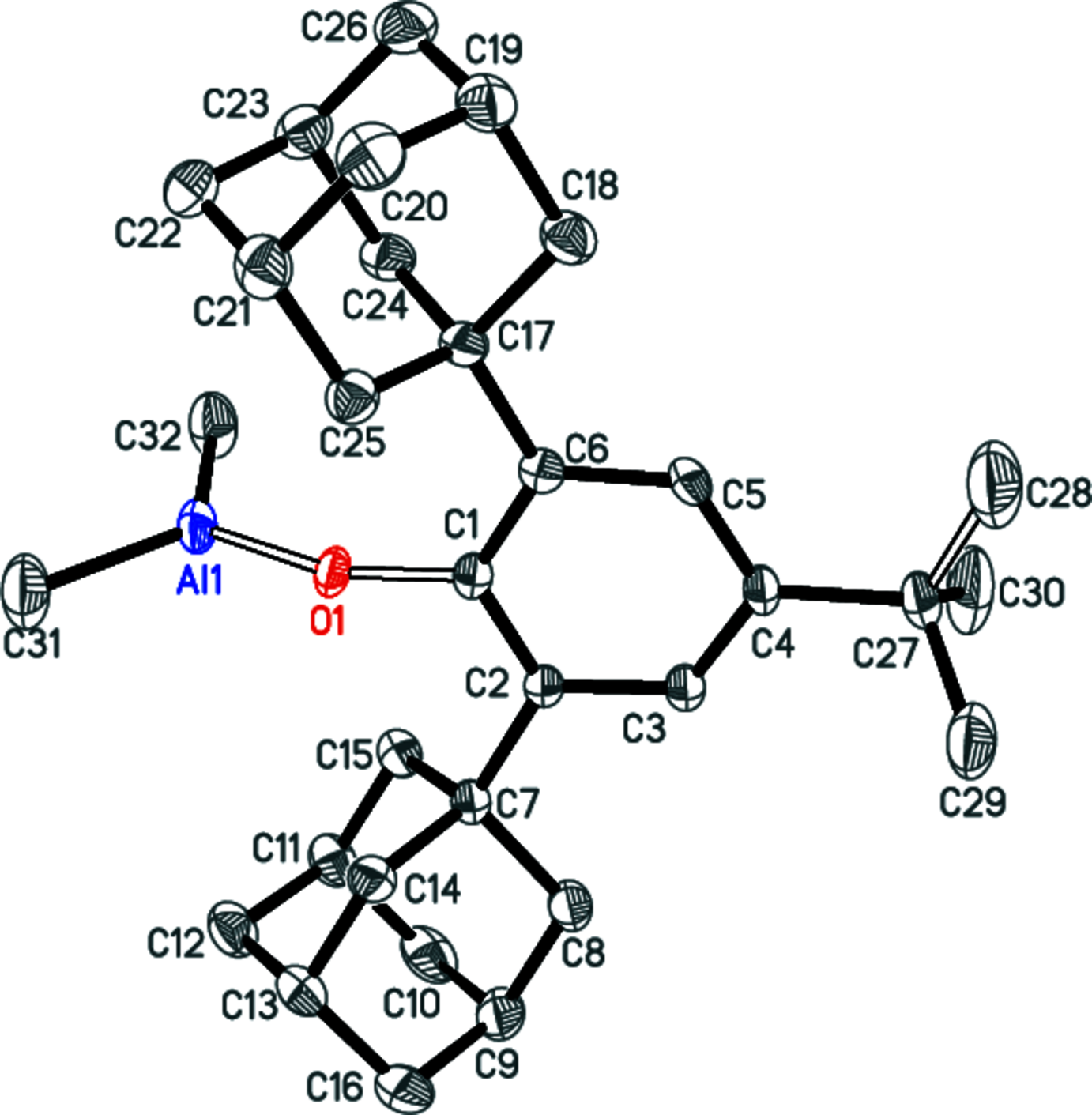

Supplement: Supplementary file 3 [file e-70-0m352-fig1.tif]

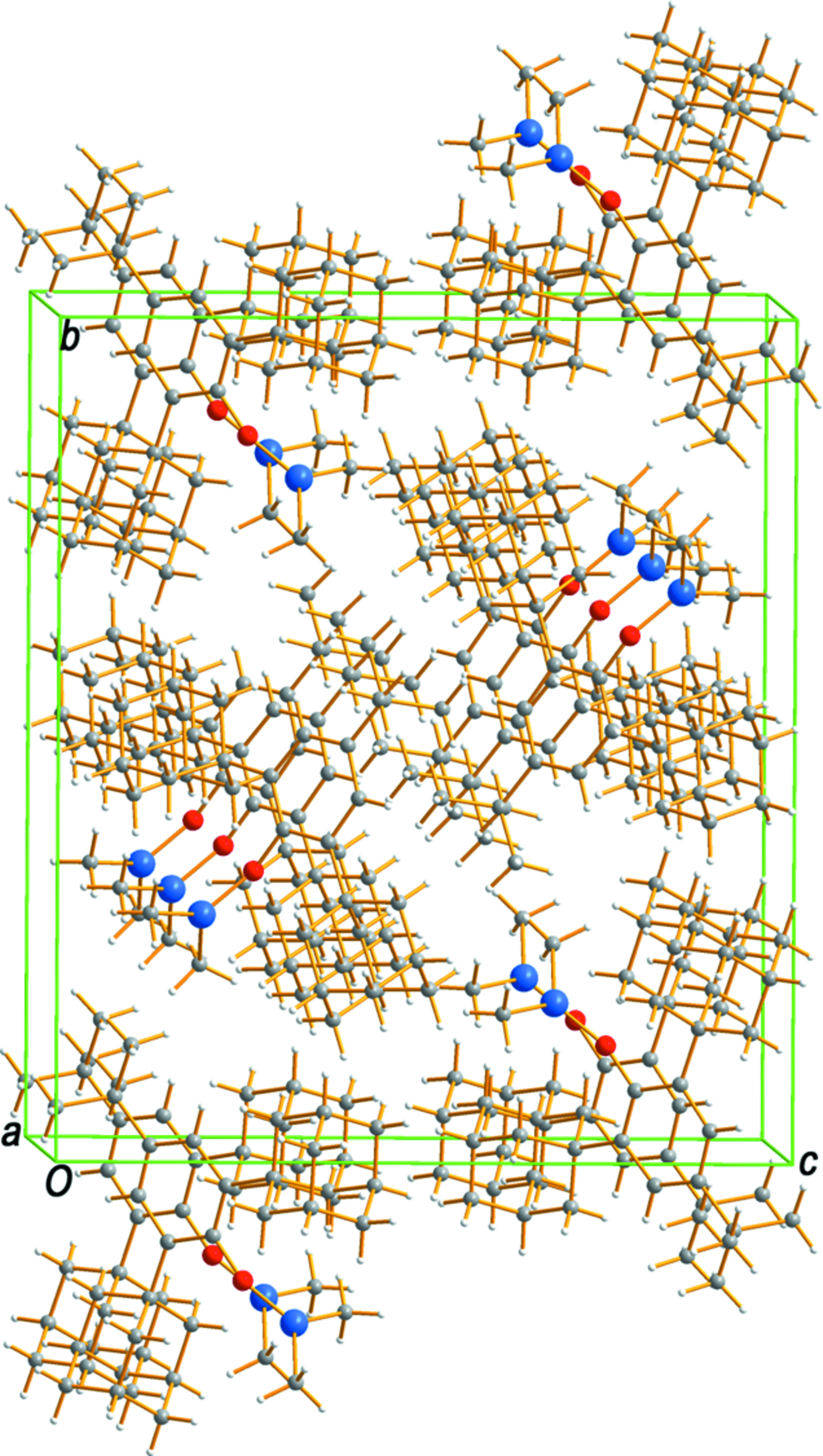

Supplement: Supplementary file 4 [file e-70-0m352-fig2.tif]
